# Neuropsychiatric Disorders Due to Limbic Encephalitis: Immunologic Aspect

**DOI:** 10.3390/ijms22010389

**Published:** 2020-12-31

**Authors:** Yu-Chia Kao, Ming-I Lin, Wen-Chin Weng, Wang-Tso Lee

**Affiliations:** 1 Department of Pediatrics, E-Da Hospital, Kaohsiung 82445, Taiwan; yukanomail2006@yahoo.com.tw; 2 Department of Pediatrics, Shin Kong Wu Ho-Su Memorial Hospital, Taipei 11101, Taiwan; m000743@ms.skh.org.tw; 3 Department of Pediatrics, National Taiwan University Hospital, Taipei 100226, Taiwan; wcweng@ntu.edu.tw; 4 Department of Pediatrics, National Taiwan University College of Medicine, Taipei 100233, Taiwan; 5 Graduate Institute of Brain and Mind Sciences, National Taiwan University College of Medicine, Taipei 100233, Taiwan

**Keywords:** limbic encephalitis, neuropsychiatric, neuronal cell-surface antibody, onconeural antibody, paraneoplastic neurologic syndrome

## Abstract

Limbic encephalitis (LE) is a rare cause of encephalitis presenting as an acute and subacute onset of neuropsychiatric manifestations, particularly with memory deficits and confusion as core features, along with seizure occurrence, movement disorders, or autonomic dysfunctions. LE is caused by neuronal antibodies targeting the cellular surface, synaptic, and intracellular antigens, which alter the synaptic transmission, especially in the limbic area. Immunologic mechanisms involve antibodies, complements, or T-cell-mediated immune responses in different degree according to different autoantibodies. Sensitive cerebrospinal fluid markers of LE are unavailable, and radiographic findings may not reveal a typical mesiotemporal involvement at neurologic presentations; therefore, a high clinical index of suspicions is pivotal, and a neuronal antibody testing is necessary to make early diagnosis. Some patients have concomitant tumors, causing paraneoplastic LE; therefore, tumor survey and treatment are required in addition to immunotherapy. In this study, a review on the molecular and immunologic aspects of LE was conducted to gain awareness of its peculiarity, which we found quite different from our knowledge on traditional psychiatric illness.

## 1. Introduction

Limbic encephalitis (LE) is an inflammatory encephalitis involving the limbic system, which encompasses the medial temporal lobe, hippocampus, and frontobasal and cingulate cortex [1]. LE is frequently associated with antibodies against the neuronal cell surface, synaptic vesicles, and intracellular proteins; therefore, it belongs to the autoimmune encephalitis (AE) category. Triggering factors include tumors, viral infections, or immune check point inhibitors (ICI’s) [2,3]. LE most often occurs in adults older than 45 years but can affect people of all ages. In addition, gender predominance varies with the type of antibody.

The clinical hallmark of LE includes acute and subacute onset of memory and cognitive deficits. Other symptoms include confusion, psychiatric symptoms (such as anxiety, depression, or psychosis), behavioral changes, seizures, movement disorders (such as ataxia, dystonia, or myoclonus), autonomic disturbances, and sleep disturbances [1,4]. The amygdala is a core region of the limbic system involved in the control of positive and negative affect, modulation of memory, and social and cognitive functions as well as behavioral adaptation to stress [5], which explains the core neuropsychiatric manifestations of LE. The basolateral complex of the amygdala is the main input site for sensory information from the thalamus and cortical regions, which plays a central role in seizure generation, particularly in the temporal lobe epilepsy [6].

According to Graus et al., the diagnosis of LE requires four criteria: (1) subacute onset (<3 months) of cognitive deficits, seizures, or psychiatric symptoms; (2) brain abnormalities in the medial temporal lobes on T2-weighted and fluid-attenuated inversion recovery magnetic resonance imaging (FLAIR MRI) images; (3) cerebrospinal fluid (CSF) pleocytosis (>5 cells per mm^3^) or an electroencephalography (EEG) showing epileptic discharges or slow-wave activity involving the temporal lobes; and (4) exclusion of alternative causes [1]. With time, the radiographic features of swelling and hyperintensity in the limbic structures may progress to mesiotemporal atrophy [7]. Some cases showed abnormalities in the basal ganglia, particularly those with leucine-rich glioma inactivated 1 (LGI1) antibody presenting with faciobrachial dystonic seizures [8,9]. In some cases, MRI may show only unilateral limbic or extralimbic involvement, and even normal finding, making the diagnosis a challenge. Fluorodeoxyglucose (FDG)-positron emission tomography (PET) is more sensitive than EEG and MRI in detecting abnormalities in LE, particularly in LGI1 and contactin-associated protein-like 2 (CASPR2) encephalitis [9,10]. A positive neuronal antibody is frequent but not mandatory for diagnosis as around 7–26% of LE cases had no detectable antibodies and are referred to as seronegative [11]. Positive antibody results are useful in diagnosing patients with atypical features or those who do not fulfill the Graus’s criteria [1].

## 2. Antibodies

Antibodies associated with LE are thought to be of peripheral origin that penetrates a leaky blood–brain barrier (BBB) or to be synthesized intrathecally. Most AE has higher serum than CSF antibody levels, implicating that antibodies are likely to be initiated by a peripheral immune response [12]. The autoantibodies are predominantly of IgG1 subclass. However, LGI1 and CASPR2 antibodies are mainly IgG4 subclass [13,14]. IgG4 antibody is hetero-bispecific (continuously undergoing half antibody exchange), hence less effective than IgG1 in crosslinking and internalizing the target antigen and does not fix complement [15]. In general, IgG1-associated AE (e.g., N-methyl-D-asparate receptor [NMDAR], γ-aminobutyric acid-B receptor [GABA-BR], and α-amino-3-hydroxy-5-methyl-4-isoxazolepropionate receptor [AMPAR]) have more inflammatory signs than IgG4-associated AE (e.g., LGI1 and CASPR2) [16]. Nevertheless, complement-mediated neuronal loss was still observed in LGI1 and CASPR2-associated neurologic syndromes [17,18]. These patients had higher complement-fixing IgG1 subclass with more prolonged clinical course, more severe cognitive impairment, and frequent hippocampal atrophy in advanced stages of the disease [13,19].

**Figure 1:**
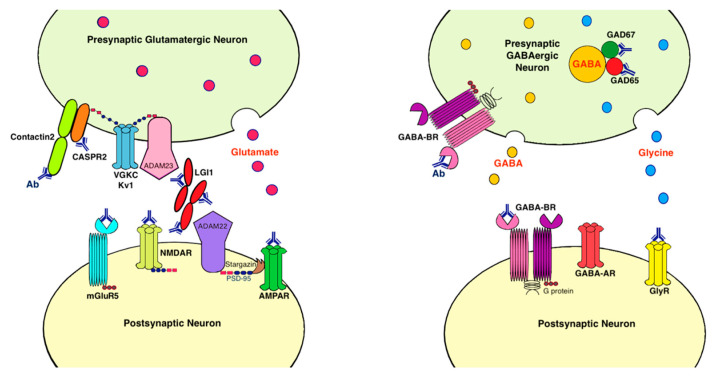
Antibodies and their pathogenic effects in limbic encephalitis. The pathologic effects of antibodies include blocking of receptors or ion channels, disruption of interaction with neighboring molecules, and crosslinking and internalization of receptors from cell surface. (1) CASPR2 and contactin2 antibodies inhibit the interaction between these proteins and reduce the clustering and surface expression of VGKC; (2) LGI1 antibody disrupts interaction between protein components such as LGI1 to ADAM22/23, downregulates VGKC and reduces AMPAR clustering and synaptic transmission; (3) AMPAR antibody causes cross-linking and receptor internalization; (4) NMDAR* antibody causes cross-linking and receptor internalization; (5) mGluR5 antibody causes decreased synaptic mGluR5 cluster density; (6) GAD antibodies target mostly the GAD65 subunits but also the GAD67 subunits, disrupting GABAergic signaling; (7) GABA-BR antibody prevents ligand binding to the receptor and alters receptor function; (8) GlyR antibody probably acts as antagonist to disrupt receptor function. Ab: antibody; ADAM: a *disintegrin* and metalloproteinase; AMPAR: α-amino-3-hydroxy-5-methyl-4-isoxazolepropionate receptor; CASPR2: contactin associated protein-like 2; GABA-AR: γ-aminobutyric acid-A receptor; GABA-BR: γ-aminobutyric acid-B receptor, GAD: glutamic acid decarboxylase; GlyR: glycine receptor; LGI1: leucine-rich, glioma inactivated 1; mGluR5: metabotropic glutamate receptor 5; NMDAR: N-methyl-D-asparate receptor; PSD-95: postsynaptic density protein 95; VGKC: voltage-gated potassium channels.

Antibodies associated with LE target: (1) cell-surface receptors (GABA, AMPA, and glycine receptors); (2) ion channels (voltage-gated potassium channels [VGKC]); (3) neighboring proteins that stabilize channel complex into the membrane (LGI1 and CASPR2); (4) enzymes that catalyze the formation of neurotransmitters glutamic acid decarboxylase (GAD); and (5) intracellular proteins (Hu, Ma2, collapsin response-mediator protein-5 [CRMP5], and amphiphysin) [20]. These antibodies are classified as neuronal cell surface, synaptic vesicle, and intracellular antibodies according to the location of their targeting antigens (Table 1). Antibodies against intracellular antigens are also known as onconeural antibodies because of their large association with tumors, and cause paraneoplastic syndromes (PNS) [21]. Pathologic effects include direct blockage of receptors and ion channels, indirect disruption with neighboring molecules interaction, and crosslinking and internalization of receptors to deplete them from the cell surface (Figure 1).

Antibodies may activate complements and natural-killer (NK) cells to induce complement-dependent cytolysis or antibody-dependent cell-mediated cytotoxicity, resulting in neuronal death. The cell-surface and synaptic antibodies alter neurotransmission leading to neuronal dysfunction, whereas intracellular antibodies are epiphenomenon of T-cell-mediated immune response rather than direct pathogenic mediators of neurological diseases. Intracellular (onconeural) antibodies are considered biomarkers for the presence of tumors [22,23]; therefore, aggressive tumor survey is necessary. Removal of tumors is mandatory to ameliorate PNS and enhance treatment response. Nevertheless, intracellular antibodies can be detected in patients without identifiable tumors or with cancer without PNS [24].

LE with cell-surface and synaptic antibodies can also be triggered by cancer, although this association has lower frequency. The most common antibodies affecting the limbic system are antibodies against LGI1, GAD, GABA-BR, AMPAR, CASPR2, Ma2, and Hu [11,25]. A latest systemic review comprising 24 studies with 263 Asian patients with LE showed that 53% had LGI1, 43% had GABA-BR, and 4% had CASPR2 antibodies [26]. About 7% of patients with LE were seronegative [11]. Rarely, ≥2 autoantibodies coexist in the same patient. Patients with LE having double or triple antibodies have worse outcomes than those with single antibody because of a higher chance of concomitant tumors [27,28,29].

In addition, patients with LE may have other comorbid autoimmune diseases, which should also be evaluated and treated. For example, about half of patients with Hashimoto encephalopathy had anti-NH2-terminal of α-enolase antibodies, and some manifested as LE [30].

## 3. Immunopathology

LE with antibodies against cell-surface antigens act mainly through antibody and/or complement-mediated mechanisms. However, LE with antibodies against intracellular antigens is predominantly mediated by cluster of differentiation (CD) 8+ cytotoxic T cells with frequent neuronal loss [17,25]. T cells are the major immune cells in the CSF, which play a major immunosurveillance role in the central nervous system (CNS) [31]. In all AE cases, CD3+ lymphocytes comprised the majority of inflammatory cells within the brain. A significant difference in the proportion of parenchymal CD8+ T cells (CD8/CD3 ratio) between intracellular and surface antibodies (mean 75% vs. 43%, respectively) was noted. The percentage of CD8+ T cells in the GAD antibody-mediated disease is intermediate (54%) [17]. The CD8+ T cells cause impairment of neuronal excitability and integrity with neuronal degeneration via two independent pathways: (1) granule cytotoxicity by perforin and granzyme-B and (2) ligation of death receptors [32,33]. Perforin is a Ca^2+^-dependent protein, which can form transmembranous pores, alter electrical excitability and signaling, and cause neuronal necrosis with swelling and rupture of cell membranes. Perforin mediates the delivery of granzymes into the target cells to promote apoptosis [34]. Apposition of multiple granzyme-B lymphocytes to single neurons, which is consistent with a specific cytotoxic T-cell attack, was observed in several onconeural antibody-mediated diseases. Therefore, higher CD8/CD3 ratio and more frequent appositions of granzyme-B+ cytotoxic T cells to neurons were associated with greater neuronal loss [17]. Cytotoxic T cells can also liberate neurotoxic cytokines, such as interferon-γ (IFN- γ) and tumor necrosis factor-α as well as excitotoxic glutamate [35]. CD8+ T cells also destroy the myelin sheath or glial cells in both white and gray matter [32].

In addition to antibodies and lymphocytes, microglia and astrocytes also play roles in mediating neuronal damages in the hippocampus and other brain regions. Microglia and astrocytes are required for synaptic integrity structurally and synaptic transmission functionally [36]. Activated microglia have dual functions, either anti-inflammatory or pro-inflammatory [37]. Previous studies showed that activated microglia in hippocampus may be associated with chronic epilepsy and was also found in VGKC-associated LE [38,39,40]. Immunotherapy may decrease the activation of microglia leading to improvement in seizure. Astrocytes and their autoimmune glial fibrillary acidic protein (GFAP) have also been shown to be associated with AE including NMDAR encephalitis and other encephalitis mimicking LE [41,42], and play important roles in neuroinflammation. The activation of astrocytes may trigger an astrocyte-neuron signaling cascade leading to persistent functional change in hippocampal excitatory synapses [43], which may be associated with cognitive impairment.

## 4. Inflammatory Mediators

Conventional CSF inflammatory markers such as white blood cells or protein are neither sensitive nor specific for LE, although the detection of oligoclonal band may increase diagnostic sensitivity [44]. A lower CD4/CD8+ T-cell ratio is detected in the CSF of all patients with LE [45].

The serum and CSF levels of C-X-C motif chemokine ligand 13 (CXCL13) were significantly higher in patients with LGI1 encephalitis [46]. The serum levels of CXCL10 were elevated in CASPR2 encephalitis. CXCL10 is a cytokine that recruits C-X-C motif chemokine receptor 3 cells such as activated T cells [47]. CASPR2 encephalitis seems to elicit a higher immune response than LGI1 encephalitis, with more intrathecal IgG and higher cytokine levels, indicated by higher CXCL13 and soluble intercellular adhesion molecule-1 (sICAM1) in the CSF. CXCL13 points to a B-cell mediated, whereas sICAM1 to a T-cell mediated neuroinflammation [48]. Patients with PNS also had high levels of chemokine CXCL10 in the CSF with the presence of IFN-γ receptors on their T cells [47].

CSF mediators are different in infectious and immune-mediated encephalitis [49]. CSF cytokines IL-21 and IFN-γ-induced protein 10 kDa (IP10) are promising in differentiating between viral encephalitis and AE [50]. IL-21 causes autoantibody production, which downregulates regulatory T cells leading to enhanced autoimmunity and increased CD8+ T cells and NK cells in AE [51], whereas IP10/CXCL10 is secreted in response to IFN-γ, which is produced as part of the Th1 in response to viral infection [52].

## 5. Genetic Factors

Human leukocyte antigen (HLA) is the main genetic factor related to autoimmunity. Anti-LGI1 encephalitis is strongly associated with HLA class II allele DRB1*07:01, which was carried by approximately 90% of patients, as well as B*44:03 and C*07:06 in the HLA class I allele [53,54]. DRB1*11:01 was detected in approximately 50% of the patients with CASPR2-antibody syndrome [54]. Anti-GAD neurological syndromes have frequent association with HLA class II haplotype DQA1*05:01-DQB1*02:01-DRB1*03:01 [55].

## 6. Cancers

Cancers occur in 20–60% of patients with LE [11,56]. LE can be classified as paraneoplastic or nonparaneoplastic, based on the presence or absence of an underlying malignancy [57].

Genetic alterations in tumor cells may trigger immune tolerance breakdown with initiation of PNS [58]. In PNS, the target antigens such as Hu, Ma, Yo, Ri, CRMP5, amphiphysin, and SOX-1 are proteins normally expressed by neurons and expressed ectopically by tumor cells. These antigens are recognized by onconeuronal antibodies or presented to T cells, which result in both cellular and humoral immune responses against neural structures [3,59]. The most common PNSs are LE and cerebellar degeneration, each accounting for approximately one-third of all PNSs [60]. Lung cancer, particularly small cell lung cancer (SCLC), testicular germ-cell tumor, thymoma, and lymphoma are the most frequent associated tumors [60,61,62]. Almost all patients with LE caused by Hu antibodies have SCLC [22]. Approximately 60% of patients with neurological syndromes associated with GABA-BR antibodies have underlying SCLC, and 60% of those with AMPAR antibodies have non-SCLC, breast cancer, or thymic tumors [63]. The presence of tumor is rare in patients with VGKC antibodies, especially LGI1 antibody, but is more common in patients with peripheral nervous system involvement such as Morvan syndrome or neuromyotonia caused by CASPR2 antibody. In the latter condition, a thymoma is common [64,65]. Therefore, screening for malignancy is usually required in patients with LE because the coexistence of tumors and their treatment influence the clinical outcome [61]. If initial tumor screening is negative, total-body FDG-PET is recommended [16], or screening can be repeated after 3–6 months, followed by screenings every 6 months for 4 years [66].

## 7. Infection

Occurrence of AE during the convalescence phase of viral CNS infections, particular NMDAR encephalitis after herpes simplex encephalitis (HSE), has been well-known [67]. Patients with HSE may also develop antibodies to GABA-BR, AMPAR, dopamine-2 receptors, or concurrently to NMDAR and LGI1, and manifest relapses of neurologic symptoms [2,68,69]. Proposed mechanisms include (1) induction of self-immunization due to a viral-induced inflammatory response leading to the release of neural tissue epitopes such as NMDAR and (2) molecular mimicry based on sequence homology or structural similarities between viral protein and neural tissue epitopes [20,70]. Human herpesvirus 6 was reported as a cause of LE in immunocompromised hosts after hematopoietic stem cell transplant [71] or triggering factor of GABA-BR encephalitis [2].

## 8. Immune Checkpoint Inhibitors

In addition to developing PNS, patients with cancers receiving biological cancer therapies with ICI’s are at risk for developing peripheral and CNS complications [72,73]. The main ICI’s are ipilimumab targeting the cytotoxic T-lymphocyte antigen 4 and pembrolizumab and nivolumab inhibiting the programmed cell death 1 pathway [74]. LE is the most frequent CNS complication of ICI’s [73]. Antibody-positive (CASPR2, Hu, Ma2, and GAD65 antibodies) or antibody-negative LE may develop weeks to 1 year after starting ICI’s therapy [75,76,77,78]. Anti-Ma2 antibody is the most common antibody found in ICI-induced LE, which carries a poor outcome [73].

## 9. Antigens Associated with LE

### 9.1. Voltage-Gated Potassium Channel Complex (VGKC)

VGKCs are located on the neuronal membranes of both the central and peripheral nervous system. The VGKC complex is composed of Kv1 subunits, which function with other extracellular proteins including LGI1, CASPR2, and contactin 2, membranous proteins a disintegrin and metalloproteinase 22 and 23 (ADAM22 and 23), and intracellular scaffolding proteins including postsynaptic density protein 95 (PSD-95) [79]. VGKC antibody is categorized into four subgroups: LGI1 positive, CASPR2 positive, VGKC-positive group lacking both LGI1 and CASPR2 antibodies (double-negative VGKC) [19], and rarely double positive group [8]. The extracellular proteins LGI1 and CASPR2 are direct antigenic epitopes of VGKC antibodies, whereas in double-negative VGKC, antibody may target another extracellular protein, the contactin-2 or intracellular epitopes [80]. VGKC antibodies cause VGKC hyperexcitability spectrum, including LE, Morvan syndrome, neuromyotonia (Isaacs’ syndrome), seizures, sleep disturbance, and autonomic dysfunction [81,82]. Patients with VGKC complex LE were more likely to have an autoimmune inflammation than patients with other syndromes [83], and may show perivascular lymphocytic infiltration and neuronal loss predominantly in the hippocampus and amygdala [38]. A complement-mediated neuronal death may play a prominent role [17]. A positive VGKC antibody, especially if tested by radioimmunoprecipitation assay, has uncertain pathogenicity and limited clinical specificity [8,84]. Testing LGI1 and CASPR2 antibodies separately is advocated instead of testing VGKC antibody alone [85].

### 9.2. Leucine-Rich, Glioma Inactivated 1 Protein (LGI1)

LGI1 is a secreted synaptic protein, which interacts with presynaptic and postsynaptic ADAM to form a trans-synaptic linkage between presynaptic ADAM23-Kv1 channel and postsynaptic ADAM22-PSD-95. LGI1 is important for AMPAR-mediated synaptic transmission [86]. LGI1 is located mainly in the hippocampus and temporal cortex, and the LGI1-ADAM22-AMPAR interaction influences both long-term depression (LTD) and long-term potentiation (LTP) [87]. LGI1 secreted from the excitatory pyramidal neurons contributes to LGI1-related epileptogenesis [88]. LGI1 antibodies inhibit binding of LGI1 to ADAM22/23, downregulate Kv1.1, reduce AMPAR clustering, and alter synaptic excitability, plasticity, and memory [89,90]. Faciobrachial dystonic seizures are very specific for LGI1-encephalitis, but only present in about 50% of patients [91]. One-third of patients had peripheral nervous system manifestations, commonly with pain and dysautonomia [8]. Comorbid tumors (mainly thymoma and SCLC) [92] occurred in <10% of patients with anti-LGI1 LE.

### 9.3. Contactin-Associated Protein-Like 2 (CASPR2)

CASPR2 is a transmembrane cell adhesion protein of the neurexin family that is located at the juxtaparanodal region of the nodes of Ranvier in myelinated axons of the central and peripheral nervous system. CASPR2 interacts with dimerized contactin-2 (also known as transient axonal glycoprotein-1) [93,94] and colocalizes with VGKC Kv1.1 and Kv1.2 [95]. CASPR2 antibodies do not reduce CASPR2 expression but inhibit the interaction of CASPR2 with its binding partner contactin 2 [96] and reduce the clustering and surface expression of Kv1, thereby interfering with axonal excitability and action potential conduction [93,97]. CASPR2 antibodies did not cause evident neuronal loss, except for a modest Purkinje cell loss in the cerebellum [12].

CASPR2 antibody-associated diseases almost exclusively affect older males. 80% of patients have neuropsychiatric features such as cognitive deficits, and 50% developed seizures. Many others presented with neuropathic pain associated with peripheral neuronal hyperexcitability such as myokymia, fasciculations, and muscle cramps [19,98]. Other syndromes include autonomic dysfunction, cerebellar symptoms, insomnia, or weight loss [19]. Cancer is more frequent in CASPR2 compared to LGI1-antibody positive patients, more commonly with thymoma [8].

### 9.4. AMPA Receptor

AMPARs are glutamatergic ionotropic transmembrane receptors composed of four subunits (GluA1–4) [99]. AMPARs are concentrated in the hippocampal CA3–CA1 region, subiculum, basal ganglia, cerebellum, and the cerebral cortex. They mediate most of the fast excitatory synaptic transmission in the brain and are critical for synaptic plasticity, learning, and memory [100]. LE patients’ antibodies usually target GluA1 or GluA2 subunits of AMPARs, which decrease AMPAR clustering at synapses through increased internalization of receptors but do not alter the synaptic density or cell viability [101,102]. In accordance with the decrease in AMPA-mediated excitatory postsynaptic currants, affected neurons also exhibit homeostatic decrease in inhibitory postsynaptic currants and reduced inhibitory synapse density, whereas the intrinsic neuronal excitability increases [102]. AMPAR antibodies do not cause significant neuronal apoptosis. Synaptic AMPAR cluster density was restored within a few days after removal of patients’ antibodies, and patients usually recovered to baseline after treatment [101].

In AMPAR encephalitis, confusion is the most frequent manifestation. Seizure occurs in approximately one-third of patients. Associated cancers were found in approximately 50–60% of patients, most commonly lung carcinoma and thymoma [27,103].

### 9.5. Metabotropic Glutamate Receptor 5 (mGluR5)

Glutamate receptors (GluRs) are the main excitatory synaptic transmission mediators in the brain. GluRs can be divided into ligand-gated ion channels (ionotropic) and G protein-coupled (metabotropic) GluRs (mGluRs). In the three groups of mGluRs, mGluR5 belongs to group I. mGluR5 are expressed primarily in the hippocampus and amygdala, which modulates LTD and LTP [104]. mGluR5 antibodies cause a decrease of mGluR5 cluster density at both synaptic and extrasynaptic locations [105]

All patients with anti-mGluR5 encephalitis had CSF pleocytosis and 75% had oligoclonal bands. MRI abnormalities revealed not only the limbic system involvement, but also the involvement of extralimbic regions such as thalamus, pons, frontal or parieto-occipital cortex, and cerebellum [105]. mGluR5 antibody is associated with LE and Hodgkin lymphoma, presented as Ophelia syndrome in nearly 50% of patients [105,106].

### 9.6. Dipeptidyl Peptidase-Like Protein 6 (DPP6)/DPPX

DPP6 (DPPX) is a membrane glycoprotein and an auxiliary subunit of the voltage-gated A-type (rapidly inactivating) potassium channel Kv4.2 [107]. Kv4.2 is involved in somatodendritic signal integration and attenuation of back-propagation of action potentials [107], which is responsible for transient, inhibitory currents in the peripheral and CNS [108]. DPPX is predominantly expressed in the hippocampus, cerebellum, and striatum [109]. DPPX antibodies are predominantly of IgG4 subtype. Loss of DPPX modulation of potassium currents leads to neuronal hyperexcitability [108].

Most patients with DPPX encephalitis had a combination of LE, brainstem dysfunction, severe diarrhea, and weight loss [107,110,111]. Many patients have characteristic gastrointestinal symptoms even before neuropsychiatric symptoms [111] because of the enriched expression of DPPX in myenteric plexus [112]. The course of neuropsychiatric symptoms in anti-DPPX encephalitis was prolonged, and chronic immunotherapy is often required [107].

### 9.7. GABA-B Receptor

GABA-BRs are transmembrane G-protein-coupled receptors composed of two subunits, the GABA-B1 and GABA-B2, and mediate slow and prolonged inhibitory neurotransmission [113]. GABA-BRs are ubiquitously distributed in the brain and spinal cord, but the highest levels are found in the hippocampus, thalamus, and cerebellum [114]. Most GABA-BR antibodies recognized the GABA-B1 subunit. GABA-BR antibodies do not decrease the synaptic levels of receptors but alter their synaptic function.

Co-expression of GABA-BR with other autoantibodies is relatively common [63,115,116,117,118]. Reported coexisting antibodies in GABA-BR encephalitis included potassium channel tetramerization domain containing 16 (KCTD16), GAD65, Hu, Ri, amphiphysin, SOX1, CV2/CRMP, 5N-type or P/Q-type voltage-gated calcium channels, or thyroid peroxidase [115,116,117,119,120]. Most of these cases were paraneoplastic.

Anti-GABA-BR encephalitis have highest rates for pleocytosis, protein concentration, and positive oligoclonal bands compared to other LE [121]. Increased CSF glutamate levels were found in GABA-BR associated encephalitis [122]. Increased CD138+ and CD19+ plasma cells and activated cytotoxic T cells in CSF corresponded to higher overall neuropsychological and memory deficits in patients with GABA-BR LE. A hippocampal specimen also revealed perivascular infiltrates of CD138+ plasma cells and CD4+ T cells, whereas cytotoxic CD8+ T cells were detected within the brain parenchyma in close contact to neurons [123].

Patients with GABA-BR LE had seizure, confusion, and memory deficits. 80–100% of patients presented an early or prominent seizure, and a large proportion had convulsive or nonconvulsive status epilepticus. Seizure was often of temporal lobe onset, usually refractory to medications but was well responsive to immunotherapy [63,115,116,120]. The seizure phase was followed by an encephalitic phase compatible with LE such as confusion and memory deficits. More than 50% of patients had tumors, mostly SCLC [115,116,124].

### 9.8. Glutamic Acid Decarboxylase (GAD)

GAD is a rate-limiting enzyme in presynaptic inhibitory neurons for synthesis of the inhibitory neurotransmitter GABA from glutamate, using pyridoxal-5′-phosphate as cofactors. GAD is widely distributed within the CNS and pancreas [125]. There are two isoforms: cytoplasmatic, a constitutively active isoform of 67 kDa (GAD67) for maintaining steady basal production of GABA and synaptic membrane-associated form of 65 kDa (GAD65), which undergoes auto-inactivation during enzyme activity and provides pulse production when rapid surge of GABA is required [126]. GAD65 is localized predominantly in nerve terminals, anchored to the cytoplasm-facing side of synaptic vesicles [127]. Compared with GAD67, GAD65 has greater antigenicity partly due to its surface electrostatic charge and greater mobility in the C-terminal and catalytic domains [127]. GAD is mostly found intracellularly but transiently exposed extracellularly during the dynamic process of neurotransmission and exocytosis [128].

GAD antibodies disrupt GABAergic signaling. GAD65 antibodies are detected in about 8% of general population, but always at low level [129]. GAD antibodies are frequently found in patients with type 1 diabetes mellitus (DM1) (80–90%), and around 40% of patients had been diagnosed with DM1 before the onset of neurologic symptoms [125,130]. Patients with neurologic syndromes associated with GAD usually have serum titers several hundred-fold higher than those with DM1 [131]. GAD antibodies from patients with DM1 did not react with brain tissue [132]. In rats, in vivo injection of GAD65 antibodies from patients with stiff-person syndrome (SPS) induced electrophysiologic changes in myelinated neurons, whereas GAD65 antibodies from patients with DM1 did not [133]. Antibodies seem to recognize different GAD65 epitopes in patients with DM1 and neurologic syndromes [134]. Pathological specimens showed T-cell infiltrates but no IgG deposition as well as acute necrosis and neuronal loss in the hippocampus, evolving to hippocampal atrophy [17,135]. Loss of GABAergic neurons in the brainstem has been observed [136].

Most patients harboring GAD65 antibodies are women, with a mean age of 23 years, which is younger compared to LE caused by other antibodies. According to one small pediatric case series of LE, 50% of patients had GAD antibodies [137]. Neurological syndromes associated with GAD antibodies include SPS, cerebellar ataxia, limbic and extralimbic encephalitis, nystagmus/oculomotor dysfunction, drug-resistant epilepsy, paraneoplastic SPS, and progressive encephalopathy with rigidity and myoclonus. The latter two are mainly related to amphiphysin and glycine receptor antibodies, respectively [138]. Seizures occurred in almost all patients with GAD-related LE, may present as status epilepticus, and were difficult to control even with immunotherapy [139,140,141].

GAD antibodies were detected in 7–17% of adult patients with LE [135]. Previous study reported a probable <15% cancer association in GAD antibody-associated LE [135]. GAD antibody-associated LE is ten times more likely to be paraneoplastic than other neurologic syndromes such as SPS or cerebellar ataxia. The most frequent tumors are lung and thymic neoplasms. Patients with PNS associated with GAD were older, male predominance, having coexisting antibodies than non-neoplastic GAD cases [116,119,142]. Cancer risk is seven times higher in patients with GAD and coexisting antibodies against neuronal cell-surface antigens, especially GABA-BR [119]. The prognosis varies and is considered intermediate between that of neurologic syndromes associated with antibodies against cell-surface and intracellular antibodies [138]. Approximately 70% of patients with high GAD65 antibody concentration improved partially after immunotherapy; however, none reached a complete recovery [130].

## 10. Intracellular Antibodies

Intracellular antigens such as Ma, Hu, Ri, Yo, CRMP5, amphiphysin, or SOX1 are considered inaccessible by their antibodies [143]. Intracellular antibodies are usually considered onconeural antibodies due to their strong association with tumors. Anti-Hu antibodies were the most frequently detected antibodies in PNS associated with onconeural antibodies. Cerebellar degeneration was the most frequent syndrome in PNS associated with onconeural antibodies, whereas brainstem encephalitis and subacute sensorimotor neuronopathy were the most frequent nonclassical syndromes [144]. LE is a rare neurologic presentation in this entity. Onconeural antibodies associated with paraneoplastic LE are Ma, Hu, Ri, CRMP5, and SOX1 [66,145], with Ma2 being the major intracellular antibody causing paraneoplastic LE [146]. In paraneoplastic LE associated with lung cancer, Hu and cell surface GABA-BR antibodies were the most frequently encountered antibodies, whereas in paraneoplastic LE associated with testicular tumor, Ma2 antibody is the most frequent antibody [147,148].

## 11. Treatment 

### 11.1. First-Line Treatment

First-line treatment (Table 2) includes pulse therapy with intravenous methylprednisolone, intravenous immunoglobulin (IVIg), or plasmapheresis. Methylprednisolone is usually given first, followed by either IVIg or plasmapheresis within 3–7 days depending on clinical features and disease severity [16].

Although IVIg and plasmapheresis neutralize or eliminate systemic antibodies, they do not target plasma cells or intrathecal antibody synthesis. Both plasma exchange (PE) and immunoadsorption (IA) resulted in clinical improvement in 60–67% in patients with AE associated with NMDAR, LGI1, CASPR2, mGluR5, GAD, and Hu antibodies [149,150,151]. Plasmapheresis was more effective for encephalitis caused by cell-surface antigens (approximately 85%) than by intracellular or synaptic antigens (66.7%) [149,150,151]. Response rate was higher with early initiation of therapy [152].

In patients with encephalitis after ICI’s treatment, early immunotherapy with steroids or IVIg is essential [153].

### 11.2. Second-Line Treatment

Second-line treatment includes rituximab, cyclophosphamide, azathioprine, mycophenolate mofetil (MMF), or others. It is usually started within approximately 2 weeks when the first-line therapies fail to achieve improvement [19,154], or when the disease is severe or relapsing.

Rituximab and cyclophosphamide are the preferred second-line treatment. Rituximab is a monoclonal antibody targeting the CD20 expressed by B cells and plasmablasts. The lifetime of antibody-producing plasma cells is typically days to weeks [155], which accounts for the delayed response of rituximab [156]. In LE with common relapses such as AMPAR encephalitis, administration of second-line therapy resulted in less relapse [157]. Encephalitis caused by DPPX usually require rituximab [111].

Cyclophosphamide, a deoxyribonucleic acid alkylating agent, targets hematopoietic cells [158], which can cross the BBB and affects both B and T cells as well as the intrathecal antibody synthesis. In GAD-associated refractory status epilepticus, cyclophosphamide resulted in seizure control and decreased intrathecal production of antibody [159]. Both rituximab and cyclophosphamide target the antibody source, causing fewer relapses, and can be combined in most severe cases. However, combination therapy carries risks of progressive multifocal leukoencephalopathy or reactivation of infections such as hepatitis B [160].

Tocilizumab is an anti-IL-6 receptor monoclonal antibody. IL-6 is a key mediator for the survival of plasmablasts and plasma cells. IL-6 not only induces B-cell differentiation and proliferation, but also promotes the differentiation of IL-17-producing helper T cells and cytotoxic T cells [161,162]. For patients with AE having inadequate clinical response to the first-line therapy and rituximab, tocilizumab had been used with favorable outcome. Nearly 90% of the tocilizumab-treated patients maintained a long-term favorable clinical response [163]. Tocilizumab also resulted in improvement in LE cases caused by CASPR2 antibodies [164] and in pediatric case of AE caused by GAD antibodies refractory to first-line treatment [165].

Daratumumab, an anti-CD38 antibody that depletes plasma cells and modifies various T-cell functions, was used in a refractory case of CASPR2 encephalitis. Daratumumab resulted in antibody titers reduction, T-cell activation markers normalization, and neurological improvement. However, the reported patient died of septicemia [166].

Bortezomib, a proteasome inhibitor that interferes with NF-κB and ubiquitin-proteasome pathway to deplete plasma cells, is currently under clinical trial for patients with severe AE (NCT03993262).

### 11.3. Maintenance Therapy

Very limited evidence on the efficacy of long-term therapy were reported. In cases unresponsive to first or second-line immunotherapy, or in cases with relapses requiring maintenance therapy, drugs such as azathioprine, **mycophenolate mofetil** (MMF), or methotrexate can be utilized [167].

Monthly intravenous or oral steroid pulses, oral steroids with tapering dose, monthly IVIg, or rituximab redosing could also be tried [168]. In patients with LE having high relapse rates such as those with LGI1 and CASPR2 antibodies, a gradual corticosteroid tapering over around 18 months can decrease relapses [169].

In general, LE associated with cell-surface antibodies are more likely to respond to immunotherapy, resulting in good recovery in up to 70–80% of cases [56]. In PNS caused by onconeural antibodies, the tissue damage is thought to be primarily cell-mediated; therefore, immunotherapy often has only little effect. The most important treatment is prompt and complete removal of tumor in order to withdraw the auto-antigen expressed by the tumor that triggers the production of autoantibodies [61]. In treatment-resistant cases, gradual neuronal loss and brain atrophy were noted [25]. For these cases, if initial tumor screening is negative, tumor surveillance must be continued at follow-up, particularly depending on the antibody [16]. In addition to intracellular antibodies, certain cell-surface antibodies (e.g., AMPAR, GABA-BR, and mGluR5) are more often associated with tumors. If antibodies are not found and the clinical presentation fulfills the criteria of LE, screening with computed tomography of the thorax is recommended, followed by a total-body FDG-PET [11]. In LGI1 patients, seizure including FBDS usually improved after immunotherapy. However, a large proportion (60%) of GAD-LE patients was not seizure free.

## 12. Prognosis

The general mortality rate of LE was 9.7%, largely due to pulmonary infection or status epilepticus. GABA-BR antibody was attributed to the highest mortality rate (23.2%), largely due to tumor progression [26]. Cognitive dysfunction is a major complication in patients with long-term LE, and some patients are left disabled [56]. In LE caused by GAD, LGI1, or CASRP2 antibodies, the memory outcomes were poor even after clinical recovery with immunotherapy. Pathologic tearfulness was reported in 50% of patients, which correlated with changes in certain emotional brain networks, although not part of the neuropsychiatric comorbidities [170].

## 13. Conclusions

**Figure 2:**
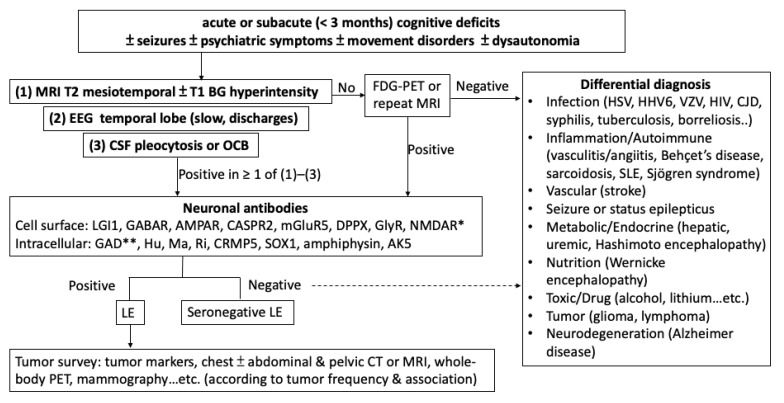
Diagnostic algorithm of limbic encephalitis. NMDAR antibody (*) generally causes autoimmune encephalitis but rarely causes limbic encephalitis. Nevertheless, it should be included in neuronal antibody survey for limbic encephalitis due to clinical similarity. GAD (**) is a synaptic enzyme.

The majority of patients with LE have prominent neuropsychiatric symptoms at disease onset, which is quite common in clinical practice. Therefore, patients with acute or subacute neuropsychiatric symptoms with the core symptom of memory deficits, particularly if associated with seizures or abnormal signal changes on brain MRI, particularly in the mesiotemporal region, should be highly suspected for LE (Figure 2 and Table 3). A diagnosis of LE is extremely important because timely immunotherapy is even more important than symptomatic therapies with antipsychotic or antiepileptic drugs, because partial or complete remission or even cure can be achieved.

## Figures and Tables

**Table 1:** Autoantibodies associated with limbic encephalitis.

	Symptoms	Tumor Association	Image	Prognosis
Cell-surface				
LGI1	LE, seizure including FBDS, hyponatremia, paroxysmal dizziness spells, pain, dysautonomia	<10% SCLC, thymoma	Normal or nonspecific changes; T2-mesiotemporal hyperintensity; T1-bright basal ganglia in FBDS	Almost fair response to immunotherapy but high relapse rate (15–35%)
CASPR2	LE, Morvan syndrome, neuromyotonia, seizure, neuropathic pain, dysautonomia	10–40% (44% for LGI1 and CASPR2 dual seropositivity) thymoma	Normal or nonspecific changes; T2 mesiotemporal hyperintensity	Favorable response to immunotherapy but high relapse rate (35%)
AMPAR	LE, confusion, psychiatric symptoms, seizure	50–70% lung, breast, thymoma	T2 mesiotemporal hyperintensity	Favorable response to immunotherapy but common relapse
GABA-BR	LE, seizure, dysautonomia, movement disorder, rapidly progressive dementia	50–60% SCLC	T2 mesiotemporal hyperintensity	Frequent co-expression with other Abs; poor prognosis with concurrent tumor or convulsive SE; relapse rate (20%)
mGluR5	LE, Ophelia syndrome seizures, movement disorders	50% Hodgkin lymphoma	Limbic and extra-limbic (thalamus, pons, frontal or parieto-occipital cortex, cerebellum)	Complete or partial recovery to immunotherapy
DPPX	LE, BE, diarrhea, CNS hyperexcitability, PERM, dysautonomia	<10–30% B cell tumor	Normal or nonspecific T2/FLAIR white matter abnormalities	Chronic and second-line immunotherapy frequently required, relapse rate (23%)
Synaptic				
GAD	LE, SPS, PERM, seizure, CA, oculomotor dysfunction, diabetes	<15% (higher with coexisting Abs, esp. GABA-BR Ab) lung, thymoma	T2 mesiotemporal hyperintensity	70% had partial improvement with immunotherapy
Intracellular				
Ma	LE, BE, diencephalic encephalitis, seizure, CS	90% testicular, lung and pleural	Nonspecific	Favorable in anti-Ma2 but poorer in anti-Ma
Hu	LE, CS, BE, dysautonomia, sensory neuropathy	>90% SCLC	Nonspecific	Poor
Ri	LE, CS, BE, OMS, movement disorders	>90% woman-breast, man-lung and bladder	Nonspecific	Common co-expression with other Abs
CRMP5	LE, encephalomyelitis, CS, SPS	>90% SCLC, thymoma	Normal or T2 mesiotemporal hyperintensity	Poor
SOX1	LE, LEMS, CS, neuropathy, LEMS	30–60% SCLC	Normal or T2 mesiotemporal hyperintensity	Common co-expression with other Abs; poor response to treatment
AK5	LE	No data	Hippocampal atrophy	Poor response to treatment

Abs: antibodies; AK5: adenylate kinase 5; AMPAR: α-amino-3-hydroxy-5-methyl-4-isoxazolepropionate receptor; BE: brainstem encephalitis; CASPR2: contactin-associated protein-like 2; CRMP5: collapsin response-mediator protein-5; CNS: central nervous system; CS: cerebellar syndrome including paraneoplastic cerebellar degeneration; DPPX: dipeptidyl peptidase-like protein 6; FBDS: faciobrachial dystonic seizures; GABA-BR: γ-aminobutyric acid B receptor; GAD: glutamic acid decarboxylase; LE: limbic encephalitis; LEMS: Lambert-Eaton myasthenic syndrome; LGI1: leucine-rich, glioma inactivated 1; mGluR5: metabotropic glutamate receptor subtype 5; OMS: opsoclonus-myoclonus syndrome; PERM: progressive encephalomyelitis with rigidity and myoclonus; SCLC: small-cell lung cancer; SE: status epilepticus; SOX1: sex-determining region Y (SRY)-box 1; SPS: stiff-person syndrome.

**Table 2:** Immunotherapy for limbic encephalitis.

	Dose & Duration	Mechanism	Note
First Line			
Methylprednisolone	Adult: 1 g daily Children: 30 mg/kg/day (max. 1 g) for 3–5 days	Inhibits NF-κB → anti-inflammation	30 mg/kg/day (max. 1 g) once monthly for 3–6 months
IVIg	2 g/kg in 2 or 5 days	Neutralizes Abs and cytokines, decreases B cells, inhibits complement activation, modulates regulatory T cells	1 g/kg monthly for 3 months or longer Should not be given immediately prior to plasmapheresis
Plasmapheresis (PE or IA)	5–7 exchanges in 10–14 days	Remove Ab	
Second Line			
Rituximab	375 mg/m^2^ weekly for 4 weeks, or 750 mg/m^2^ (max. 1000 mg/dose) for two doses 2 weeks apart	Anti-CD20 Ab → depletion of B cells and plasmablasts	
Cyclophosphamide	750–1000 mg/m^2^ (max. 1000–1500 mg/dose) monthly for 3–6 months	Akylating agents inhibiting DNA synthesis → suppress B and T cells	Cause infertility in repeated doses
Tocilizumab	8–12 mg/kg (max. 800 mg) monthly for 6 months	Anti-IL6 receptor Ab → inhibits B and T cells	
Daratumumab	16 mg/kg weekly in cycle 1–8, every two weeks in cycle 9–13	anti-CD38 Ab → depletion of plasma cells	One case of anti-CASPR2 encephalitis
Bortezomib	1.3 mg/m^2^ on day 1, 4, 8, and 11 of a 21-day cycle, total 3 cycles	proteasome inhibitor → depletion of plasma cells	Clinical trial (NCT03993262)
Maintenance therapy			
Prednisolone	1–2 mg/kg/day once daily or divided for 4 weeks; tapered over several weeks to months	Inhibits NF-κB → anti-inflammation	
Mycophenolate mofetil (MMF)	Initial 300 mg/m^2^/day, target 600 mg/m^2^/day, 1–1.5 g/day, twice daily	Inhibits purine nucleotides → inhibits B and T cells	
Azathioprine (AZA)	1–2.5 mg/kg/day (max.150 mg/day), once daily	Inhibits purine synthesis → inhibits B and T cells	
Methotrexate (MTX)	Oral: 10 mg/m^2^ weekly Intrathecal: 10 mg weekly for 4 weeks	Inhibits NF-κB → anti-inflammation	

**Table 3:** Characteristic symptoms and laboratory findings of limbic encephalitis.

**Symptoms**	**Antibodies**
Seizure	GABA-BR (~100%), LGI1 (80–100%), GAD (60–100%), CASPR2 (75%), NMDAR*(70%), AMPAR (33%) FBDS in LGI1 (33–67%)
Paroxysmal dizzy spells	LGI1 (14%)
Cramps or neuropathic pain	CASPR2, LGI1
Movement disorders	NMDAR *, CRMP5, Hu, GlyR, GABA-BR, DPPX, CASPR2, Ri
Dysautonomia	NMDAR *, LGI1, CASPR2, GABA-BR, DPPX, GlyR
GI symptoms (diarrhea)	DPPX
**Laboratory findings**	**Antibodies**
Hyponatremia	GLI1 (~50%), CASPR2 (~25%)
Hyperglycemia	GAD
CSF pleocytosis + OCB	GABA-BR, GAD, mGluR5

NMDAR antibody (*), a frequently detected antibody in autoimmune encephalitis, should also be included in antibody survey for limbic encephalitis due to clinical similarity. AMPAR: α-amino-3-hydroxy-5-methyl-4-isoxazolepropionic acid receptor; CASPR2: contactin-associated protein-like 2; CSF: cerebrospinal fluid; DPPX: dipeptidyl peptidase-like protein 6; FBDS: faciobrachial dystonic seizures; GABA-BR: γ-aminobutyric acid B receptor; GAD: glutamic acid decarboxylase; GI: gastrointestinal; GlyR: glycine receptor; LGI1: leucine-rich, glioma inactivated 1; NMDAR: N-methyl-D-aspartate receptor; OCB: oligoclonal band.

## Data Availability

Data sharing not applicable.

## Abbreviations

AE	autoimmune encephalitis
AK5	adenylate kinase 5
AMPAR	α-amino-3-hydroxy-5-methyl-4-isoxazolepropionic acid receptor
BBB	blood-brain barrier
BG	basal ganglia
CASPR2	contactin-associated protein-like 2
CJD	Creutzfeldt-Jakob disease
CNS	central nervous system
CRMP5	collapsin response-mediator protein-5
CSF	cerebrospinal fluid
DPPX/DPP6	dipeptidyl peptidase-like protein 6
EEG	electroencephalography
FDG-PET	fluorodeoxyglucose-positron emission tomography
FLAIR	fluid-attenuated inversion recovery
GABA-BR	γ-aminobutyric acid B receptor
GAD	glutamic acid decarboxylase
GlyR	glycine receptor
HIV	human immunodeficiency virus
HSV	herpes simplex virus
HHV6	human herpesvirus 6
ICI	immune checkpoint inhibitor
IL	interleukin
INF-γ	interferon-γ
LE	limbic encephalitis
LGI1	leucine-rich, glioma inactivated 1
mGluR5	metabotropic glutamate receptor subtype 5
MRI	magnetic resonance imaging
NK	natural killer
NMDAR	N-methyl-D-aspartate receptor
OCB	oligoclonal band
PNS	paraneoplastic syndrome
SLE	systemic lupus erythematosus
SOX1	sex-determining region Y (SRY)-box 1
SPS	stiff-person syndrome
VZV	varicella zoster virus
